# p120 catenin recruits HPV to γ-secretase to promote virus infection

**DOI:** 10.1371/journal.ppat.1008946

**Published:** 2020-10-21

**Authors:** Mara Calypso Harwood, Allison Jade Dupzyk, Takamasa Inoue, Daniel DiMaio, Billy Tsai

**Affiliations:** 1 Department of Cell and Developmental Biology, University of Michigan Medical School, Ann Arbor, MI, United States of America; 2 Cellular and Molecular Biology Program, University of Michigan Medical School, Ann Arbor, MI, United States of America; 3 Department of Microbiology and Immunology, Stanford University School of Medicine, Stanford, CA, United States of America; 4 Pathogen Research Section, Central Research Laboratory, Research and Development Division, Japan Blood Products Organization, Kobe, Japan; 5 Department of Genetics, Yale School of Medicine, New Haven, CT, United States of America; Johns Hopkins University, UNITED STATES

## Abstract

During internalization and trafficking, human papillomavirus (HPV) moves from the cell surface to the endosome where the transmembrane protease γ-secretase promotes insertion of the viral L2 capsid protein into the endosome membrane. Protrusion of L2 through the endosome membrane into the cytosol allows the recruitment of cytosolic host factors that target the virus to the Golgi *en route* for productive infection. How endosome-localized HPV is delivered to γ-secretase, a decisive infection step, is unclear. Here we demonstrate that cytosolic p120 catenin, likely via an unidentified transmembrane protein, interacts with HPV at early time-points during viral internalization and trafficking. In the endosome, p120 is not required for low pH-dependent disassembly of the HPV L1 capsid protein from the incoming virion. Rather, p120 is required for HPV to interact with γ-secretase–an interaction that ensures the virus is transported along a productive route. Our findings clarify an enigmatic HPV infection step and provide critical insights into HPV infection that may lead to new therapeutic strategies against HPV-induced diseases.

## Author summary

Human papillomavirus (HPV) is the primary cause of cervical, anogenital, and oropharyngeal cancers. Despite the significant impact of HPV on human health, there is limited understanding of how this small DNA virus traffics through a host cell to cause infection. This work identifies the role of the cellular factor p120 catenin in routing the virus along a productive entry pathway. Specifically, we propose that p120 targets HPV to the transmembrane protein γ-secretase, a critical step of viral entry. This work thus provides insights into the intracellular transport mechanisms of HPV and identifies a potential therapeutic target for HPV treatment.

## Introduction

Human papillomavirus (HPV) infects nearly 80 million U.S. adults [1] and is the primary cause of cervical, anogenital, and oropharyngeal cancers [2]. While efficacious prophylactic vaccines exist against 7 of the cancer-causing HPVs [2], the vaccines have not been efficiently utilized, with over half the target population remaining unvaccinated in the U.S. [3]. One consequence of underutilized HPV vaccines is the alarming increase in the number of HPV-associated oropharyngeal cancers, surpassing that of cervical cancers in the U.S. in recent years [4]. Despite HPV’s significant impact on human health, there is limited understanding of its cellular entry mechanisms leading to infection. Thus, identifying host factors essential for HPV infection may reveal novel targets for anti-viral therapy and remains an important objective in combating HPV-induced diseases.

Structurally, HPV is a nonenveloped virus composed of the viral capsid proteins L1 and L2 which encase the viral DNA genome [5]. At the plasma membrane, L1 binds to extracellular heparin sulfate proteoglycans, resulting in a series of conformational changes to the viral capsid [6–14]. The N-terminus of the L2 protein is then cleaved by furin at the cell surface [15–17] and the virus is subsequently transferred to an unknown entry receptor [10,18]. Through an unknown mechanism, the virus is then endocytosed [19–21]. Immediately after endocytosis, HPV is delivered to the endosome, where some of the L1 and L2 molecules are disassembled from the incoming virus particle [22,23].

In the endosome, the viral particle is targeted to a critical factor–the transmembrane protease γ-secretase [24–27]. In addition to its well-characterized protease function [28,29], our labs recently discovered that the catalytic subunit of γ-secretase, presenilin-1 (PS1), harbors a novel chaperone activity that promotes insertion of the C-terminus of L2 into the endosome membrane [24]. This insertion event enables this segment of L2 to protrude into the cytosol, a step mediated by a cell-penetrating peptide (CPP) on the C-terminus of the L2 protein [30,31]. Subsequently, host factors such as retromer and SNX17 are recruited to the cytosolic segment of L2, and the virus is guided to the Golgi *en route* to the nucleus for infection [32–34].

Targeting of HPV to γ-secretase for membrane insertion is a critical step because it directs HPV along an infectious route [24]. However, it is not known how HPV is targeted to γ-secretase to engage this tightly-controlled host factor [28]. One possible scenario is that HPV is recruited to γ-secretase by hijacking pre-existing “γ-secretase adaptors” [28,29]. One such factor is the cytosolic host protein p120 catenin (p120), which is known to target cell-surface transmembrane proteins to γ-secretase for proteolytic processing [35,36].

We report here that HPV interacts with cytosolic p120, likely via a transmembrane receptor, at the cell surface. Upon reaching the endosome, the virus undergoes low pH-dependent disassembly of the L1 protein in a step that does not require p120 or γ-secretase. However, p120 is essential for HPV to associate with γ-secretase. This allows for membrane insertion of L2 into the endosome membrane, a requisite step for productive trafficking. These findings provide important new insight into HPV entry, revealing how the virus hijacks γ-secretase—along with its adaptor—to promote infection.

## Results

### p120 binds to HPV16 and promotes virus infection

To study HPV internalization and trafficking, we used a well-established HPV pseudovirus (PsV) system consisting of wild-type (WT) viral capsid proteins L1 and L2 and a reporter plasmid expressing green fluorescent protein with a C-terminal S-tag (GFP-S) or secreted luciferase (Luc) in place of the viral genome (S1A Fig) [37,38]. A 3xFLAG epitope tag appended to the C-terminus of L2 (termed “L2F”) allows for detection of the virus particle throughout the trafficking events [25]. Importantly, key trafficking steps of the HPV PsV resemble that of HPV produced by stratified keratinocyte raft cultures [37]. For instance, both raft-derived virus and PsV require γ-secretase for infection [25–27].

**Fig 1:**
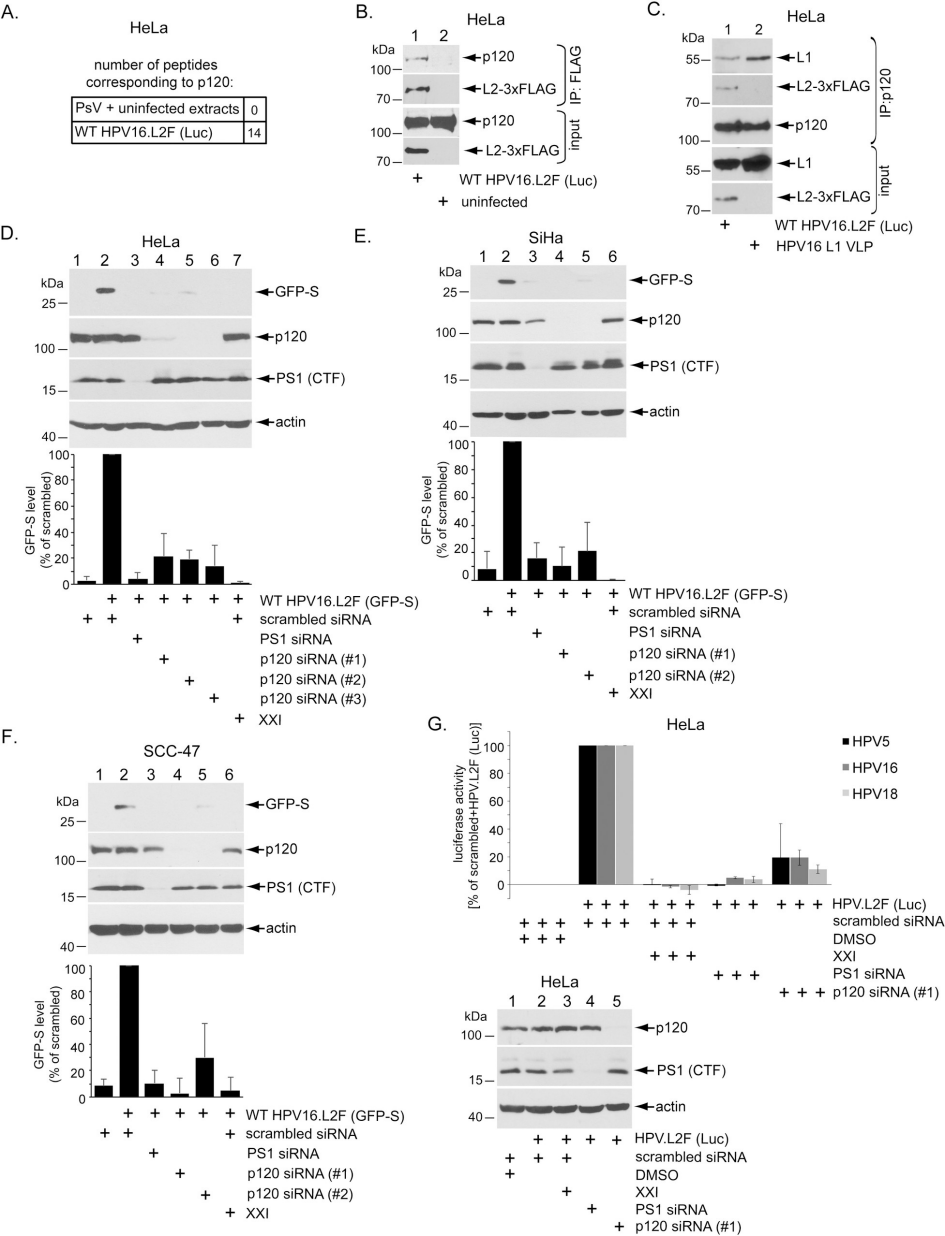
p120 binds to HPV16 and promotes virus infection. **A.** Summary of L2 immunoprecipitation-mass spectrometry performed on samples of HeLa cells infected for 16 hrs or uninfected cell extract incubated with purified HPV16 PsV as described in Inoue et al., 2018. **B.** HeLa cells infected with or without WT HPV16.L2F (Luc) for 6 hrs were lysed and the resulting extract subjected to immunoprecipitation using a FLAG antibody. The precipitated material was analyzed by SDS-PAGE and immunoblotting using the indicated antibodies. Samples labelled input were not immunoprecipitated. See S1B Fig for IgG control. **C.** HeLa cells were infected with HPV16.L2F (Luc) or HPV16 L1 VLP (virus-like particles containing L1 only). 2.5 hpi, cells were lysed and the resulting extract subjected to immunoprecipitation using an antibody against p120. The precipitated material was subjected to SDS-PAGE and immunoblotting using antibodies recognizing the indicated proteins. Samples labelled input were not immunoprecipitated. See S1C Fig for IgG control. **D.** HeLa cells transfected with the indicated siRNA were infected with WT HPV16.L2F (GFP-S), with or without γ-secretase inhibitor XXI. 48 hpi, cells were lysed and the resulting extract subjected to SDS-PAGE and immunoblotting using the indicated antibodies. Graph shows data normalized against WT HPV16.L2F (GFP-S)-infected cells treated with scrambled siRNA, and further normalized against the actin level. Data represent the mean ± SD of at least three independent experiments. **E.** As in D, except the human SiHa cervical cancer cells were used. **F.** As in D, except the human SCC-47 oropharyngeal cancer cells were used. **G.** HeLa cells transfected with the indicated siRNA were infected with HPV5, HPV16, or HPV18 pseudovirus harboring a luciferase reporter plasmid, with or without γ-secretase inhibitor XXI. 48 hpi, luciferase activity was measured from the cell culture media. Graph shows luciferase activity normalized against scrambled siRNA-treated cells with and without HPV. Data represent the mean ± SD of at least three independent experiments. The immunoblot reveals the extent of p120 (or PS1) depletion.

We recently used an immunoprecipitation-mass spectrometry (IP-MS) approach and found that WT HPV16.L2F (Luc) binds to γ-secretase during entry [24]. In addition, the cytosolic host factor p120 catenin (p120) was significantly enriched in the virally-associated proteins identified by this approach, compared to proteins pulled down from uninfected cell extracts incubated with highly purified PsV (Fig 1A); the full IP-MS data set can be found in S1 Table. Interestingly, p120 is a known binding-partner of γ-secretase [35,36]. To confirm this IP-MS result [24], we used anti-FLAG antibody to immunoprecipitate the HPV16 L2 minor capsid protein and showed by immunoblotting that p120 co-immunoprecipitated with L2 in extracts from HPV16 PsV-infected HeLa cells but not from uninfected cells (Fig 1B); as a negative control, an isotype-matched IgG control antibody did not precipitate p120 or the C-terminal fragment of the catalytic γ-secretase subunit PS1 from extracts of WT HPV16.L2F (Luc)-infected cells (S1B Fig). These results indicate that p120 is a direct or indirect binding partner of L2 during HPV infection.

We next asked if the p120-HPV16 interaction requires L2, or if L1 is sufficient to bind to p120. To test this, we used an HPV virus-like particle (VLP) containing only L1 (HPV16 L1 VLP). Immunoprecipitation of endogenous p120 co-immunoprecipitated L1 in cells infected with HPV16 L1 VLP or with WT HPV16.L2F (Luc), which contains both L1 and L2 (Fig 1C); as a negative control, an isotype-matched IgG control antibody did not pull down L1 or L2 from extracts of WT HPV16.L2F (Luc)-infected cells (S1C Fig). These results demonstrate that capsids consisting of only HPV L1 are sufficient to associate with p120. Thus, L1 may in fact mediate an indirect interaction between L2 and p120.

To probe whether p120 plays a role in HPV infection, p120 was depleted by using three unique p120-targeting siRNAs [39,40], and HPV infection was measured by immunoblotting for expression of a GFP-S reporter construct contained within the HPV16 PsV [WT HPV16.L2F (GFP-S)]. p120 knockdown was confirmed by immunoblotting. We found that p120 depletion by each of the p120 siRNAs in HeLa cells inhibited HPV infection by ~80% compared to scrambled siRNA-treated cells (Fig 1D). As expected, perturbing γ-secretase (by either knockdown of PS1 or treatment with the γ-secretase inhibitor XXI) potently blocked infection [24–27]. p120 depletion similarly inhibited HPV infection in SiHa cervical cancer cells (HPV-positive) (Fig 1E) and SCC-47 [41] oropharyngeal cancer cells (HPV-positive) (Fig 1F). Three different siRNAs were used because some cell lines were more responsive to specific siRNAs than others.

We further confirmed these results using a WT HPV16 PsV harboring a luciferase reporter plasmid [WT HPV16.L2F (Luc)], which again revealed a ~80% reduction in infection in response to p120 knockdown in HeLa cells (Fig 1G). Importantly, these p120-depleted cells were also markedly resistant to infection by HPV5 or HPV18 pseudovirus (Fig 1G); XXI treatment or PS1 knockdown also blocked HPV5 and HPV18 infection. Partial depletion of p120 in the HPV-negative C33A and HaCaT cells also decreased HPV16 PsV infection as assessed by luciferase activity (S1G and S1H Fig). As controls, we found that depletion of p120 had no effect on cell viability as measured by trypan blue staining (S1D Fig) or cell doubling time as assessed by direct counting of the cell number (S1E Fig). Furthermore, the p120-depleted cells were susceptible to infection by the polyomavirus SV40, another non-enveloped DNA virus, which does not require γ-secretase for infection (S1F Fig). These results confirm that cell toxicity or proliferation was not a confounding factor in the p120 siRNA knockdown experiments. Together, our data demonstrate that p120 promotes infection of different HPV types in multiple biologically-relevant cell-types, including the SCC-47 oropharyngeal cells previously untested for HPV infection [41].

### A γ-secretase mutant that cannot bind to p120 inefficiently supports HPV infection

**Fig 2:**
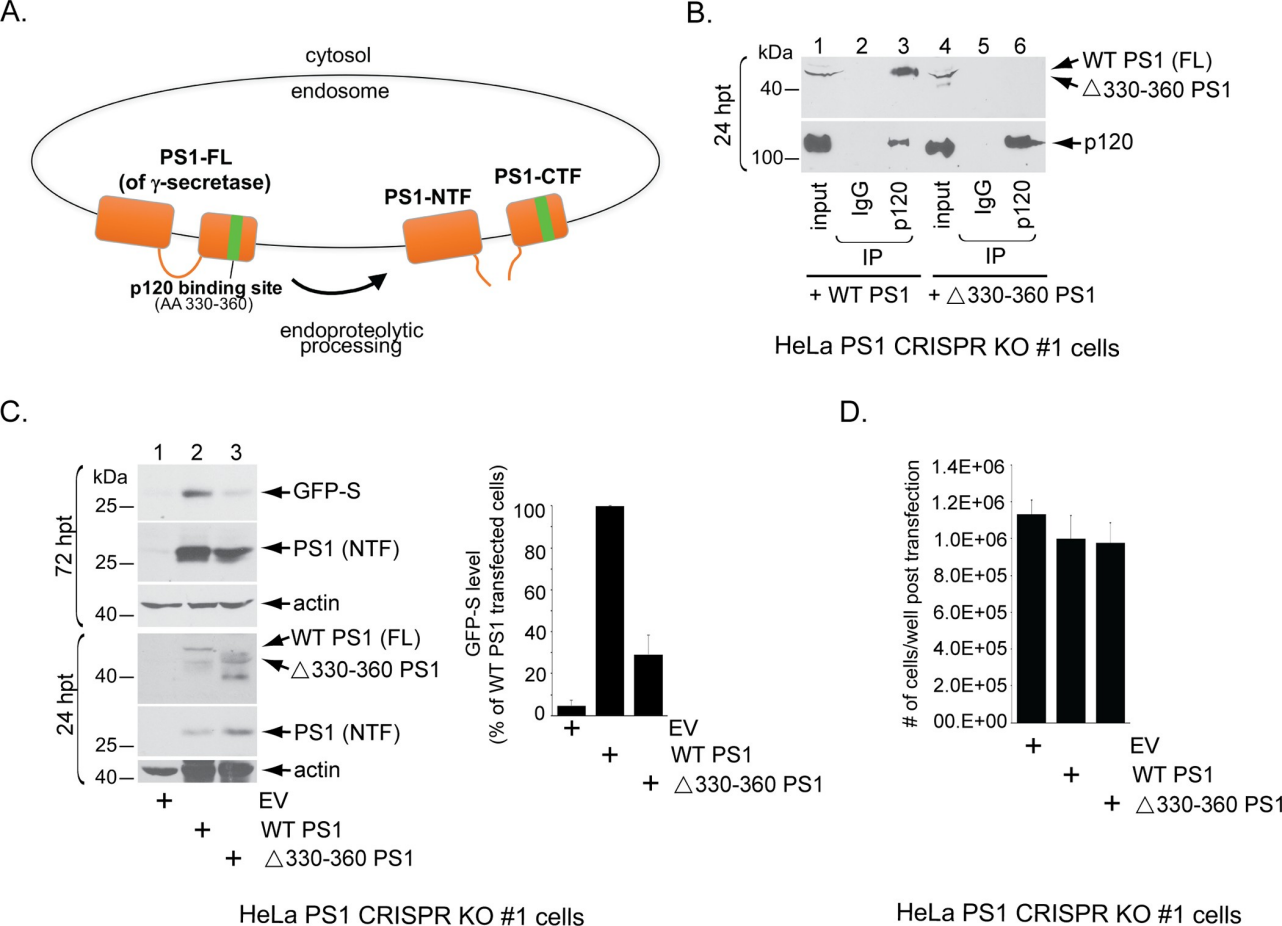
A γ-secretase mutant that cannot bind to p120 inefficiently supports HPV16 infection. **A.** A model depicting the p120-binding site in the PS1 subunit of γ-secretase. p120 binds to amino acid (AA) 330–360 of PS1. “FL”, full-length; “NTF”, N-terminal fragment; “CTF”, C-terminal fragment **B.** HeLa PS1 CRISPR knockout cells were transfected with a plasmid expressing either WT PS1 or PS1 deleted of amino acids 330–360 (Δ330–360 PS1). 24 hrs post-transfection (hpt), cells were lysed and the resulting extract was subjected to immunoprecipitation using either a control IgG antibody or an antibody against p120. The precipitated material was subjected to SDS-PAGE and immunoblotting using antibodies recognizing p120 or FLAG-tagged L2. Only the full-length (FL) PS1 (prior to endoproteolytic processing) is shown. **C.** (Upper panels, 72 hrs post-transfection, hpt) HeLa PS1 CRISPR knockout cells were transfected with empty vector (EV), WT PS1, or Δ330–360 PS1 for 24 hrs, and then infected with WT HPV16.L2F (GFP-S). 48 hpi, cells were lysed and the resulting extract subjected to SDS-PAGE and immunoblotting using the indicated antibodies. The levels of GFP-S were quantified as in Fig 1. Data are normalized against infected cells transfected with WT PS1, and further normalized against the actin levels. Data represent the mean ± SD of three independent experiments. (Lower panels, 24 hrs post-transfection, hpt) A pool of cells were harvested 24 hrs post-transfection with the indicated DNA in the absence of PsV. The resulting cell extract was subjected to SDS-PAGE and immunoblotting using antibodies recognizing the indicated proteins to visualize protein levels at the time of infection. Both the full-length PS1 and the N-terminal fragment of PS1 (due to endoproteolytic processing) are observed at the 24 hpt time point. **D.** HeLa PS1 CRISPR KO #1 cells were seeded at equal amounts in a 6-well plate and transfected with empty vector (EV), WT PS1, or Δ330–360 PS1 for 24 hrs. 72 hours after transfection, cells were harvested and the total number of cells per condition were counted by hemocytometer. Data represent the mean ± SD of three independent experiments.

p120 is an established γ-secretase adaptor, binding to the C-terminal fragment of the PS1 subunit of γ-secretase and recruiting substrates to the enzyme (Fig 2A; [28,29,35,36]). We therefore asked whether the ability of γ-secretase to bind to p120 is crucial for HPV infection. To test this, we used a γ-secretase mutant that cannot bind to p120 [35]; this mutant contains a deletion of amino acids 330–360 in the γ-secretase PS1 subunit (Δ330–360 PS1). To confirm that the PS1 mutant does not interact with p120, HeLa cells lacking endogenous PS1 due to CRISPR-mediated knockout (HeLa PS1 CRISPR KO #1 cells [24]) were transfected with WT PS1 or Δ330–360 PS1. Only the full-length PS1 is shown in the blot, although both WT PS1 and Δ330–360 PS1 can undergo endoproteolytic processing, generating an N-terminal fragment and a C-terminal fragment of PS1 [28,35]. As expected, immunoprecipitation of p120 pulled down WT PS1 (Fig 2B, lane 3) but not Δ330–360 PS1 (Fig 2B, lane 6). As a control, we found that expression of WT or mutant PS1 did not significantly affect cell doubling in the HeLa PS1 CRISPR KO cells (Fig 2D).

We then tested the ability of Δ330–360 PS1 to support HPV infection. Consistent with previous data [24], HeLa PS1 CRISPR KO cells did not support HPV infection (Fig 2C, lane 1), as assessed by expression of the GFP-S reporter protein. When WT PS1 was expressed in these cells, HPV infection was restored (Fig 2C, lane 2; quantified in right graph), as previously reported [24,25]. However, expression of Δ330–360 PS1 inefficiently restored infection, even though the mutant protein was efficiently expressed (Fig 2C, lane 3; quantified in right graph). Because this PS1 deletion mutant was previously shown to remain catalytically active [35] and can still undergo endoproteolytic cleavage into an N-terminal fragment and a C-terminal fragment, its inability to fully restore HPV infection is unlikely to be due to a global folding defect. The residual ~30% of infection that is achieved in the presence of the PS1 deletion mutant suggests that there may be a p120-independent mechanism to target HPV to PS1, possibly through other γ-secretase adaptors. These data therefore demonstrate that γ-secretase binding to p120 is required for efficient HPV infection, and along with the loss-of-function results further establish p120 as an important host factor during HPV entry.

### HPV16 binds to p120 prior to engaging γ-secretase

**Fig 3:**
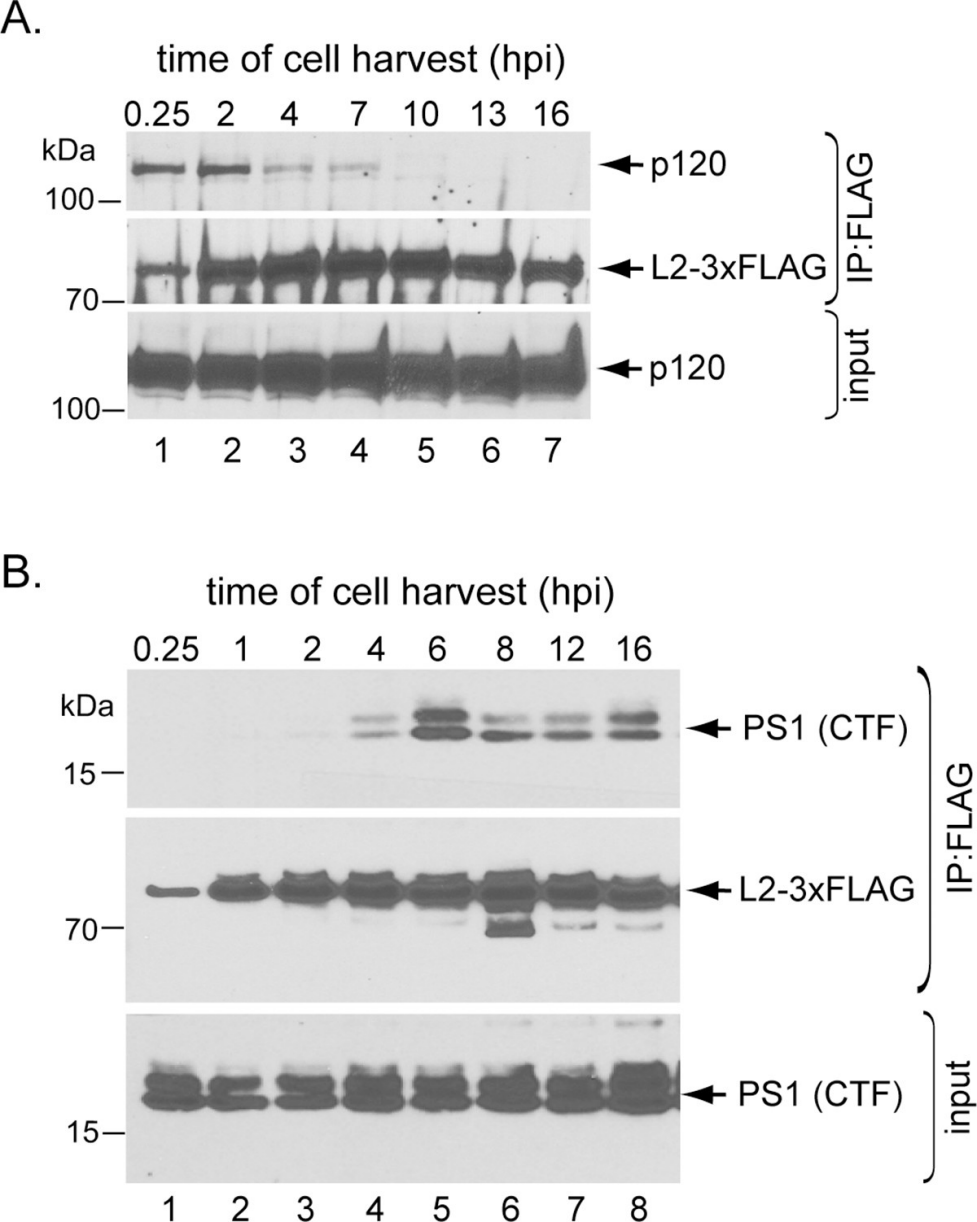
HPV16 binds to p120 prior to engaging γ-secretase. **A-B.** HeLa cells infected with HPV16.L2F (Luc) for the indicated time points were lysed and the resulting extract subjected to immunoprecipitation using FLAG antibody. The precipitated material was analyzed by SDS-PAGE and immunoblotting using antibodies recognizing p120 or FLAG-tagged L2 (panel A) or PS1 or FLAG-tagged L2 (panel B).

We next performed a time-course experiment to determine when p120 binds to HPV during entry. To this end, we infected HeLa cells with the WT HPV16.L2F (Luc) and immunoprecipitated 3xFLAG-tagged L2 at different time points across 16 hrs of infection. We found that p120 associates with HPV L2 soon after addition of virus. Maximal association occurred at 0.25–2 hrs post-infection (hpi), and this association is decreased by 4 hpi and lost by 7–10 hpi (Fig 3A). Although we initially identified p120 by using the IP-MS approach performed at 16 hpi (Fig 1A), immunoblotting did not detect HPV-associated p120 at this time point, likely because the MS approach is a much more sensitive method than immunoblotting. By contrast, association of HPV L2 to the γ-secretase PS1 subunit was first detectable at 4–6 hpi (Fig 3B), similar to a previous report [24]. These data demonstrate that HPV16 binds to p120 before the virus engages γ-secretase and that the loss of p120 binding is roughly coincident with the acquisition of γ-secretase binding. Of note is the appearance of a minor <70 kDa L2 protein species correlating with association of the virus with PS1 (Fig 3B). We have previously characterized this species to be a γ-secretase-cleaved form of the L2 protein [24] and determined that cleavage of L2 by γ-secretase is not essential for the function of γ-secretase during HPV infection.

### Low pH initiates partial L1 capsid disassembly via a p120 and γ-secretase-independent mechanism

**Fig 4:**
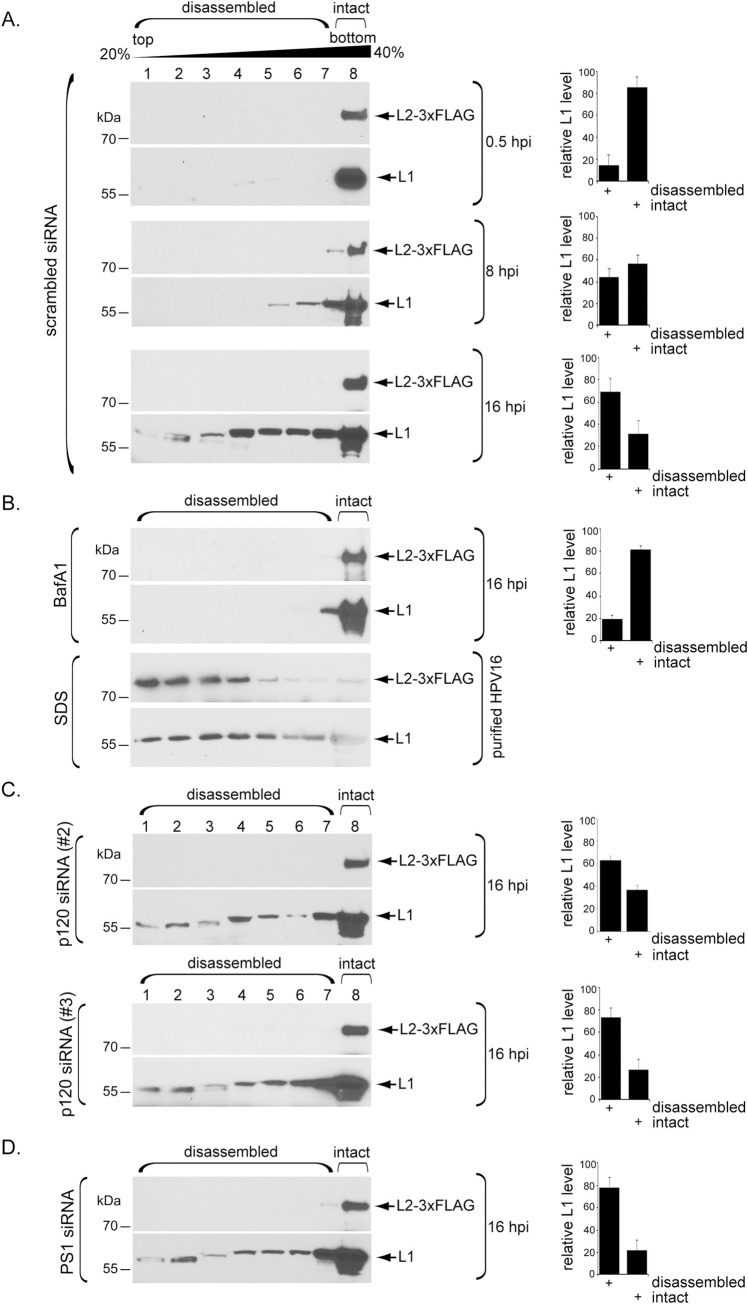
Low pH initiates partial L1 capsid disassembly via a p120- and γ-secretase-independent mechanism. **A.** HeLa cells transfected with scrambled siRNA were infected with WT HPV16.L2F (Luc). At the indicated time point post infection, cells were lysed and the resulting extract layered on top of a discontinuous 20–40% sucrose gradient. Following centrifugation, fractions were collected and subjected to SDS-PAGE and immunoblotting using antibodies recognizing L1 or FLAG-tagged L2. The levels of L1 in fractions 1–7 (disassembled) and fraction 8 (intact) were quantified. Data represent the mean ± SD of at least three independent experiments. **B.** (upper panels) HeLa cells were treated with bafilomycin A1 (BafA1) for 24 h, then infected, processed, and quantified as in A. Data represent the mean ± SD of at least three independent experiments. (lower panels) Purified WT HPV16.L2F (Luc) was treated with the chemical denaturant SDS, and the sample processed as in A. **C.** HeLa cells transfected with the indicated p120 siRNA were infected, processed, and quantified as in A. Data represent the mean ± SD of at least three independent experiments. **D.** HeLa cells transfected with the PS1 siRNA were infected, processed, and quantified as in A. Data represent the mean ± SD of at least three independent experiments.

Previous studies suggested that HPV undergoes low pH-dependent partial disassembly of L1 in the endosome during entry [19,22,23]. Our observation that p120 loses its interaction with HPV 7–10 hpi (Fig 3A), time points when HPV is in the endosome [24], prompted us to ask whether p120 might be required for HPV to undergo pH-dependent capsid disassembly. To test this, cells were infected with HPV16.L2F (Luc), harvested at various times after infection, and the resulting cell extract subjected to a virus capsid disassembly assay based on sucrose gradient centrifugation and SDS-PAGE (Fig 4).

At 0.5 hpi when HPV is bound at the cell surface primed for endocytosis, both L1 and L2 are found in the most rapidly sedimenting sucrose fraction (fraction 8), suggesting that the viral particles have not experienced significant disassembly–we refer to these viral particles in fraction 8 as largely intact (Fig 4A, 0.5 hpi, fraction 8). The L1 levels in fraction 8 representing the intact virus and fractions 1–7 representing disassembled virus are quantified in the right graph. Consistent with previously published imaging data [42–44], L2 remains associated with the viral DNA throughout infection. However, at 8 hpi when some of the virus has arrived at the endosome, a small pool of L1 sedimented more slowly (Fig 4A, 8 hpi, fractions 1–7; quantified in the right graph), indicating that a minority of molecules of L1 have disassembled from the capsid. At 16 hpi, when the bulk of the virus is endocytosed, a greater portion of L1 sedimented slowly (Fig 4A, 16 hpi, fractions 1–7; quantified in the right graph). Furthermore, when endosomal acidification was blocked by bafilomycin A1 (BafA1), L1 disassembly was significantly inhibited (Fig 4B, fraction 8; quantified in the right graph). By contrast, addition of the chemical denaturant SDS fully disrupts the virus, including dissociation of L2 which was not seen under physiological conditions (Fig 4B). While these data suggest that under normal conditions, L1 undergoes pH-dependent partial disassembly during HPV internalization and trafficking, it is evident that not all L1 proteins have dissociated from the L2-viral genome complex. Even at 16 hpi, some L1 is present in the heaviest sucrose fraction, suggesting that it remains intact with L2 and the viral genome (Fig 4A). This is consistent with reports that a portion of L1 reaches the nucleus along with L2 and the viral genome [44,45].

Importantly, upon p120 knockdown, L1 disassembly is observed at 16 hpi (Fig 4C, fractions 1–7; quantified in the right graph), indicating that p120 is not required for HPV to undergo pH-mediated disassembly. Likewise, knockdown of PS1 had no effect on virus disassembly (Fig 4D, fractions 1–7; quantified in the right graph), as expected since γ-secretase is not known to be involved in endosomal arrival of HPV. Together, these data indicate that p120 (and γ-secretase) are not required for L1 capsid disassembly. Additionally, the finding that HPV still undergoes disassembly in cells depleted of p120 (or γ-secretase) but not in cells in which endosome acidification is blocked indicate that p120 and γ-secretase do not promote initial endosome entry of HPV, despite the fact that p120 binds to HPV at early timepoints during infection. This is consistent with our earlier report that γ-secretase knockdown or inhibition do not block localization of HPV to endosomes at 8 hpi [24,25].

### p120 promotes L2 binding to γ-secretase and membrane insertion

**Fig 5:**
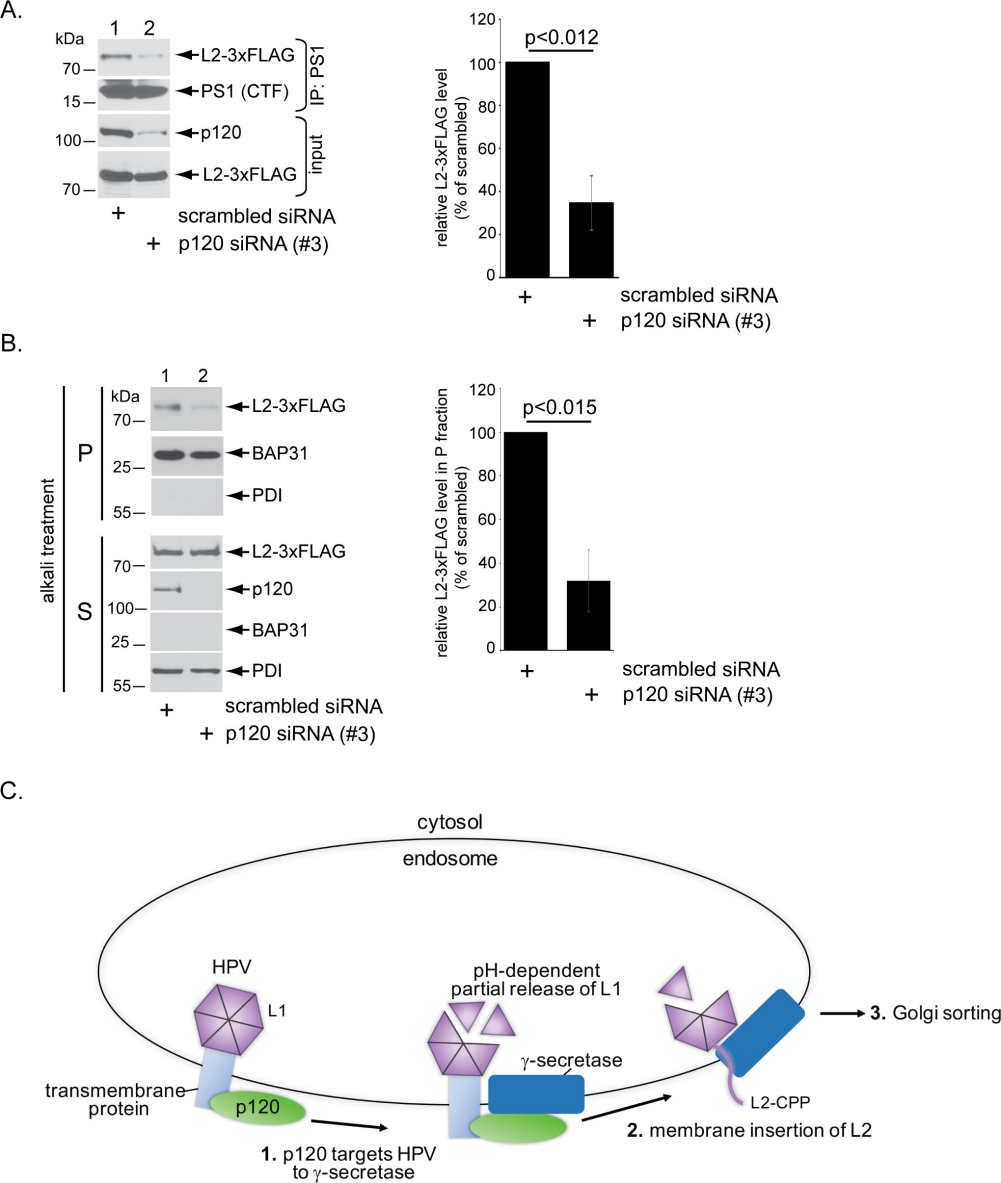
p120 promotes L2 binding to γ-secretase and membrane insertion. **A.** HeLa cells transfected with the indicated siRNA were infected with WT HPV16.L2F (Luc) for 16 hrs. Cells were lysed and the resulting extract subjected to immunoprecipitation using an antibody against PS1. The precipitated material was subjected to SDS-PAGE and immunoblotting antibodies recognizing the indicated proteins. The level of immunoprecipitated L2-3xFLAG is quantified in the right graph. Data represent the mean ± SD of at least three independent experiments. A two-tailed *t* test was used to determine statistical significance. **B.** HeLa cells infected with WT HPV16.L2F (Luc) for 16 hrs were mechanically homogenized and processed to generate a membrane fraction (see Materials and Methods), which was treated with alkali reagents and recentrifuged to generate a pellet fraction (which contains transmembrane proteins) and a supernatant fraction (which harbors soluble proteins). Both fractions were subject to SDS-PAGE and immunoblotting with antibodies recognizing the indicated proteins. Presence of the membrane protein BAP31 in the pellet fraction and soluble protein PDI in the supernatant fraction confirm proper fractionation. The level of L2-3xFLAG in the pellet fraction is quantified in the right graph. Data represent the mean ± SD of at least three independent experiments. A two-tailed *t* test was used to determine statistical significance. **C.** Proposed model for p120-dependent targeting of HPV16 to γ-secretase. In the endosome, HPV L1 partially dissociates from L2 in a pH-dependent manner. p120 bound to HPV, presumably via a transmembrane protein, targets the virus to γ-secretase (step 1). This is a critical step as γ-secretase promotes insertion of the viral capsid protein L2 into the endosome membrane (step 2). Membrane protrusion mediated by the cell-penetrating peptide (CPP) on the C-terminus of L2 then exposes the retromer binding site to the cytosol, which in turn recruits host factors that direct the virus along an infectious route through the Golgi apparatus and to the nucleus (step 3).

Although p120 is not essential for low pH-dependent disassembly of the L1 capsid protein in the endosome, p120 might play a critial role in targeting HPV to γ-secretase since p120 is known to deliver cellular substrates to γ-secretase [35,36,46]. To test whether p120 was required for assocation between HPV and γ-secretase, we depleted p120 and used coimmunoprecipitation to probe the level of HPV-γ-secretase binding. We found that knockdown of p120 disrupted the HPV-γ-secretase interaction (Fig 5A; quantified in the right graph), suggesting that p120 is required for HPV to engage γ-secretase.

When HPV is recruited to γ-secretase, the viral L2 protein is inserted into the endosome membrane via the chaperone activity of γ-secretase. Thus, we asked if p120 is essential for γ-secretase-dependent membrane insertion of L2. A robust alkali resistance assay was previously developed to monitor insertion of L2 into the endosome membrane [24]. In this approach, L2 remains in the alkali-resistant final “pellet” (P) fraction if it is inserted into the membrane. We found that upon p120 knockdown, the level of L2 in the alkali-resistant pellet fraction was markedly reduced (Fig 5B; quantified in the right graph), indicating that p120 is essential for γ-secretase to engage and insert L2 into the endosome membrane.

## Discussion

This study clarifies an important HPV entry event. Upon endosomal arrival, HPV is delivered to the transmembrane protease γ-secretase which promotes insertion of L2 into the endosome membrane and CPP-mediated protrusion of a segment of L2 into the cytosol (Fig 5C, step 2) [24,25,30,31]. The L2 C-terminus in the cytosol in turn recruits cytosolic sorting factors (such as the retromer) which delivers HPV to the Golgi *en route* for successful infection (Fig 5C, step 3) [32–34]. Without γ-secretase-induced membrane insertion of L2, the virus does not enter the retrograde transport pathway and infection cannot proceed [24]. Hence, the delivery of HPV to γ-secretase in the endosome represents a decisive HPV infection event, although how this is accomplished is unknown.

We used an unbiased IP-mass-spectrometry screen [24] to identify p120 as a host factor that binds to HPV during viral internalization and trafficking; whether the L1 and L2 viral proteins both participate in binding to p120 is unclear, but capsids consisting of L1 alone are sufficient for this interaction. We then confirmed with genetic knockdowns that p120 is essential for infection of multiple HPV types in several cell types, including oropharyngeal SCC-47 cells [41]. As the prevalence of HPV-associated oropharyngeal cancers has recently surpassed that of HPV-induced cervical cancers in the U.S. [4], the use of these cells may provide a relevant model to study HPV-associated oropharyngeal cancer.

What specific HPV entry step is mediated by p120? Our data revealed that the HPV-p120 interaction occurs early during HPV infection, prior to the virus reaching the endosome and associating with γ-secretase. Importantly, depletion of p120 blocked interaction of HPV with γ-secretase and membrane insertion of L2 by this transmembrane protease. However, depletion of p120 did not block low pH-induced disassembly of the virus in the endosome. Based on these results, we propose that p120 acts to target HPV to γ-secretase independently of capsid disassembly (Fig 5C, step 1). The observation that p120 depletion does not block L1 disassembly from the viral capsid strongly suggests that the action of p120 is not required for low pH-induced L1 disassembly. Hence, in this scenario, L1 disassembly can still continue at 16 hpi even though p120 is no longer interacting with HPV.

The model that p120 acts to target HPV to γ-secretase (Fig 5C, step 1) is in agreement with the established role of p120 in targeting cellular transmembrane proteins to γ-secretase [28,29,35,36,46]. By associating with p120, HPV simply exploits this pathway to reach γ-secretase. Our model further suggests that γ-secretase must engage p120 so that γ-secretase can capture HPV and insert the L2 protein into the endosome membrane–this idea is indeed supported by the finding that a γ-secretase mutant that cannot bind to p120 failed to efficiently promote HPV infection. However, there was some residual infection in cells expressing the p120 mutant or knocked-down for p120, suggesting the presence of a p120-independent mechanism to target HPV to PS1. For example, there may be other γ-secretase adaptors that facilitate this virus targeting step.

Because p120 is a cytosolic factor, and the non-enveloped HPV is in the endosome lumen after endocytosis and does not become exposed to the cytosol until after interaction with γ-secretase, a critical question is how p120 engages HPV before reaching γ-secretase. One possibility is that the HPV-p120 interaction is bridged by an unidentified transmembrane protein. As p120 binds to HPV early during infection, a cell surface receptor of HPV might in fact be this transmembrane protein. In this regard, several cell surface receptors including integrins, growth factor receptors, tetraspanins, and annexin A2 have been implicated as HPV receptors [7,21,47–53]; whether these receptors physically link HPV to p120 are unknown.

p120 is a well-established binding-partner of the cell-cell adhesion transmembrane protein cadherin [35,36,46,54]. Because p120 has also been shown to deliver cadherins to γ-secretase [28,29,35,36], an intriguing scenario is that HPV binds to the cadherin component of the cadherin-p120 complex at the cell surface. When p120 delivers cadherins to γ-secretase, which itself spans the membrane, HPV is concomitantly recruited to γ-secretase. However, no functional data have been reported regarding a role of cadherins during HPV infection. Clearly, identifying the transmembrane protein(s) coupling HPV to p120 will be a fruitful area for future investigation. In sum, our findings reported here provide further mechanistic insights into a critical HPV entry step, illuminating how the virus hijacks the action of an adaptor of the critical host component γ-secretase to promote infection.

## Materials and methods

### Antibodies and inhibitors

**Table:** 

Antibodies			
Antigen	Species	Source	Application
FLAG M2	Mouse mono	Sigma	WB, IP
P120	Mouse mono	Santa Cruz	WB, IP
HPV16 L1	Mouse mono	Sigma	WB
S-tag	Rabbit poly	Abcam	WB
PS1 C-terminal fragment	Rabbit mono	CST	WB
PS1 N-terminal fragment	Rabbit poly	Biolegend	WB
HSP90	Mouse mono	Santa Cruz	WB
β-actin	Rabbit mono	CST	WB
Bap31	Rat mono	Fisher	WB
PDI	Mouse mono	Abcam	WB
SV40 T Ag	Mouse mono	Santa Cruz	IF
**Inhibitors**			
**Compound**	**Solvent**	**Source**	**Concentration**
XXI	DMSO	Millipore	1 μM
Bafilomycin A1 (BafA1)	DMSO	Millipore	30 nM

IP, immunoprecipitation; WB, Western blot; IF, immunofluorescence

### DNA constructs

The p16sheLL, p5sheLL, and p18sheLL plasmids were gifts from Dr. John Schiller (National Cancer Institute, Rockville, MD; Addgene plasmids #37320, #46953, and #37321). These plasmids were used as in Zhang et al., 2014 [25] to produce the WT HPV16.L2F, HPV5.L2F, and HPV18.L2F pseudoviruses (PsVs) with FLAG tagged L2, and WT L1. The WT PS1 pCDNA3.1(-) construct used was generated in Inoue et al., 2018 [24]. Δ330–360 PS1 was subcloned from the pMX-IRES-GFP retroviral expression vector [35,55] (a gift from Dr. Nikolaos Robakis, Mount Sinai School of Medicine) and inserted into pcDNA3.1(-) for mammalian transfection.

### Cells

HeLa cells were purchased from American Type Culture Collection. The 293TT cells were a generous gift from Dr. Christopher Buck (National Cancer Institute, Rockville, MD). SCC-47 cells were a gift from Dr. Thomas Carey (University of Michigan). HeLa PS1 CRISPR KO #1 cells were generated as in Inoue et al., 2018. SiHa, C33A, and HaCaT cells were supplied by Dr. Dan DiMaio (Yale University). All cells were cultured in DMEM (Thermo Fisher Scientific) and 10% fetal bovine serum (Corning) containing 10 μg/ml streptomycin and 10 U/ml penicillin (Thermo Fisher Scientific).

### HPV pseudovirus production

WT HPV16.L2F, WT HPV18.L2F, and WT HPV5.L2F pseudoviruses were produced according to Inoue et al., 2018 [24], and as described in [56]. Briefly, 293TT cells were co-transfected with p16sheLL.L2F, p18sheLL.L2F, or p5sheLL.L2F along with indicated reporter construct (phGluc expressing secreted *Gaussia* luciferase, or pcDNA3.1 expressing GFP with a C-terminal S-tag) with polyethyleneimine (Polysciences Inc.). After 48 hrs, cells were lysed in a buffer containing 0.5% Triton X-100, 10 mM MgCl_2_, and 5 mM CaCl_2_. The lysate was incubated overnight at 37°C with 250 U/mL Benzonase Nuclease (Millipore) and 10 U/mL Plasmid-Safe DNase (Lucigen). The resulting extract was centrifuged on an OptiPrep gradient of 27, 33, and 39% at 300,000 g for 4 hrs at 16°C in a SW55 Ti rotor. Purity of the pseudovirus preparations was analyzed by subjecting the virus to SDS-PAGE and Coomassie staining (Invitrogen) (S1A Fig). We estimated the amount of L1 to be ~1000 ng/μL by performing SDS-PAGE and Coomassie blue staining on purified pseudovirus preparations and comparing the amount of L1 to protein standards electrophoresed in parallel. HPV16.L1 (L1 only VLP) was a gift from Pengwei Zhang, Yale University.

### siRNA transfection

siRNAs used in this study were generated by Sigma-Aldrich and target the following sequences:

**Table:** 

siRNA			
Name	Sequence	Conc.	Sequence source
PS1	5’-UCAAGUACCUCCCUGAAUG-3’	25-100nM	Inoue et al., JCB 2018 [24]
p120 siRNA #1	5'-AACGAGGUUAUCGCUGAGAAC-3'	100nM	Xiao et al., JCB 2003 [40]
p120 siRNA #2	5'-GCGAUUGCUUCGAAAGGCUCGUGAU-3'	100nM	Zhu et al., Journal of Cell Science, 2012 [39]
p120 siRNA #3	5’-GCUAUGAUGACCUGGAUUA-3’	100nM	predesigned by Sigma-Aldrich

As a negative control, Allstar siRNA (Qiagen) was used. HeLa cells were seeded and simultaneously reverse transfected with 100 nM siRNA using Lipofectamine RNAi MAX (Thermo Fisher Scientific) and OPTI-MEM (Gibco) and incubated for at least 48 hrs prior to infection or biochemical assays.

### Immunoprecipitation mass-spectrometry

The IP mass-spectrometry results shown in Fig 1 and S1 Table are from Inoue et al., 2018 [24]. Briefly, HeLa cells were asynchronously infected with WT HPV16.L2F at a concentration of ~12.5 pg L1/cell for 16 hrs or uninfected. The cells were harvested, and lysed in 3 mL of a buffer containing 50 mM Hepes (pH 7.5), 150 mM NaCl (HN buffer), 1% Triton X-100, and 1 mM phenylmethylsulfonyl fluoride (PMSF). Centrifugation of the resulting extract at 16,100x g generated a supernatant fraction that was incubated with FLAG M2 antibody (0.3 μg/ml) at 4°C for 2 h. The immune complex was then captured with protein G-coated magnetic beads (Thermo Fisher Scientific). The HeLa cell extract derived from uninfected cells were mixed with OptiPrep-isolated HPV16.L2F prior to incubation with FLAG M2 antibody and was used as a background control to determine post-lysis binding proteins. The bound proteins were eluted with 0.1 mg/ml 3xFLAG peptide (Sigma) and precipitated by trichloroacetic acid (TCA). The TCA-precipitated materials were subjected to mass-spectrometry analysis (Taplin Mass Spectrometry Core Facility, Harvard Medical School).

### Immunoprecipitation

HeLa cells were plated at approximately 5 x 10^6^ in 10 cm plates and incubated for 16–24 hrs (until ~80–90% confluent). Cells were then infected with WT HPV16.L2F at a concentration of ~5.7 pg L1/cell for indicated times before lysis in 1% Decyl Maltose Neopenyl Glycol (DMNG) (Anatrace) in HN buffer (50 mM Hepes + 150 mM NaCl) and 1 mM PMSF. Lysed cells were centrifuged at 16,100 g for 10 min and the resulting supernatant was incubated with antibody against M2 FLAG, PS1 C-terminal fragment, p120, or an equal concentration of IgG control antibody overnight at 4°C. Protein G magnetic Dynabeads (Thermo Fisher) were then added to the sample for 1 hr at 4°C. Beads were washed 3X in 0.1% DMNG in HN buffer, and incubated at 95°C for 10 minutes with 5X SDS sample buffer plus 2-mercaptoethanol (BME) and subjected to SDS-PAGE and immunoblotting as indicated. In Fig 1B, HeLa cells were infected with HPV16.L2F at a concentration of ~6.25 pg L1/cell for 6 hrs before treatment as above. In Fig 1C, HeLa cells were infected with HPV16.L2F or HPV16.L1 at a concentration of ~6.25 pg L1/cell for 2.5 hrs before treatment as above (L1 only VLP, provided by Pengwei Zhang, Yale University). In Fig 5A, cells were seeded and reverse transfected with the indicated siRNAs for 24 hrs and infected at a concentration of ~6.25 pg L1/cell for 16 hrs before treatment as above. In Fig 2B, HeLa PS1 CRISPR KO #1 cells were seeded in 10 cm plates. Cells were grown to ~80% confluencey (about 1 day) and transfected with 5 ug WT PS1 or Δ330–360 PS1 using polyethyleneimine (Polysciences Inc.) for 24 hrs. Immunoprecipitation was then performed as above, except that cells were lysed in 0.5% NP-40 (Sigma) in 100 mM Hepes and 1 mM PMSF, and the beads were washed in 0.05% NP-40 in 100 mM Hepes.

### HPV16 infection studies

All infections were performed asynchronously. In Fig 1D–1F, the indicated cell types were seeded with approximately 4x10^5^ cells per well in 6-well plates and reverse transfected with 100nM of the indicated siRNAs for 72 hrs, followed by infection with WT HPV16.L2F (GFP-S) at a concentration of ~4.2–8.4 pg L1/cell. Cells were treated with DMSO (Sigma) or compound XXI (1 μM) at time of infection. 48 hpi, cells were harvested in 1% Triton X-100 in HN buffer and 1 mM PMSF. Lysed cells were centrifuged at 16,100 g for 10 min and the resulting supernatant was incubated at 95°C for 10 minutes with 5X SDS sample buffer plus BME and subjected to SDS-PAGE and immunoblotting with the indicated antibodies. In Figs 1G, S1G and S1H, cells were instead infected with WT HPV16 pseudovirus containing a luciferase reporter genome (Luc) a concentration of ~4.2–8.4 pg L1/cell. 48 hpi, 20 μl of media supernatant was collected from each well and luciferase activity was measured using BioLux Gaussia Luciferase Assay Kit (NEB) according to manufacturer’s instructions. In Fig 2C, HeLa PS1 CRISPR KO #1 cells were seeded in 6-well plates. Cells were grown to ~80% confluency (about 1 day) and transfected with 2 ug of plasmid expressing WT PS1 or Δ330–360 PS1 using polyethyleneimine (Polysciences Inc.) the following day. Cells were plated in duplicate, and one set was harvested at 24 hrs post-transfection to evaluate protein expression levels. The other set of cells were infected with WT HPV16.L2F (GFP-S) and incubated for an additional 48 hrs prior to harvesting and processing for SDS-PAGE as above.

### Trypan blue assay

In S1D Fig, HeLa cells were seeded with approximately 4x10^5^ cells per well in 6-well plates and simultaneously reverse transfected with 100 nM of the indicated siRNA for 72 hrs. Cells were then trypsinized, resuspended in PBS, and diluted at a 1:1 ratio in 0.4% Trypan Blue Solution (Thermo Fisher). The solution was loaded on a hemocytometer and the number of blue-staining cells was quantified relative to the number of total cells. Three biological replicates were performed for a total of ~300 cells per condition. Bars represent the means ± SD. Statistical significance was determined by the student’s *t* test.

### Cell doubling assay

In S1E Fig, HeLa cells were seeded with approximately 4x10^5^ cells per well of a 6-well plate and simultaneously reverse transfected withs 100 nM of the indicated siRNA for 72 hrs. Cells were then trypsinized, resuspended in media, loaded on a hemocytometer, and the total number of cells per well was counted for each condition. In Fig 2C, HeLa PS1 CRIPSR KO#1 cells were seeded with 4x10^5^ cells per well of a 6-well plate. The next day, cells were transfected with 2 ug of the indicated DNA using PEI. 24 hpt, cells were trypsinized, resuspended in media, loaded on a hemocytometer, and the total number of cells per well was counted for each condition.

### SV40 infection assay

In S1F Fig, HeLa cells seeded on glass coverslips in a 6-well plate (with approximately 7.5 X 10^5^ cells per well) were simultaneously reverse transfected with the indicated siRNAs. Cells were incubated for 72 hrs, then infected with SV40 (MOI ~25). Cells were then fixed in 1% paraformaldehyde and permeabilized in 0.2% Triton X-100 in PBS, incubated with anti-SV40 T antigen primary antibody, followed by an anti-mouse 594 fluorophore. Coverslips were mounted on slides using prolong Gold plus DAPI mounting agent and cells were visualized using fluorescent microscopy. The number of cells expressing large T antigen was scored relative to the total number of cells. Approximately 500 cells were counted per condition per replicate.

### Disassembly assay

In Fig 4, HeLa cells were seeded in 6-cm plates with approximately 6.4 X 10^5^ cells per plate and simultaneously reverse transfected with indicated siRNA against p120 (100 nM), PS1 (25 nM), or with control Allstar siRNA (100 nM). After 24 hrs of siRNA-mediated knockdown, cells were infected with WT HPV16.L2F (GFP-S) at a concentration of ~6.25 pg L1/cell. Infection was allowed to proceed for the indicated time, followed by lysis in 50 μl of 1% Triton X-100 in HN buffer with 100X PMSF. Cells were lysed at 4°C for 10 min, followed by centrifugation at 4°C for 10 min at 16,100 X g. The resulting extract was layered on top of a discontinuous 20–40% sucrose gradient and centrifuged at 50,000 X g for 30 min at 4°C. After centrifugation, individual sucrose layers were collected and incubated at 95°C for 10 min with 5X SDS running buffer plus BME. Fractions were subjected to SDS-PAGE and immunoblotting as indicated. For the SDS-treated samples, purified WT HPV16L2.F (GFP-S) was treated with 1% SDS in HN buffer for 10 min at 4°C. For the BafA1-treated samples, cells were treated with 1 nM BafA1 for 2 hrs prior to 16 hr infection with WT HPV16.L2F (GFP-S). For quantification, the L1 band densities in fractions 1–7 (disassembled) or fraction 8 (intact) were divided by total amount of L1. Bars represent the means ± SD of at least three independent biological replicates.

### Alkali extraction assay

Data in Fig 5B were generated as in Inoue et al., 2018 [24]. Briefly, HeLa cells were seeded at 2.0 x 10^6^ cells in 6-cm plates, grown to 80–90% confluency (approximately 1 day), and infected with WT HPV16.L2F (GFP-S) a concentration of ~6.25 pg L1/cell for 16 hrs. After 16 hrs, cells were collected and resuspended in HN buffer before being homogenizeds in 10 μm clearance ball bearing homogenizer (Isobiotech). The cell homogenate was then centrifuged at 16,100 x g at 4°C for 10 min. The resulting supernatant was centrifuged at 50,000 rpm 4°C for 30 min in a TLA 100.3 rotor (Beckman). Pellet was treated with 25 μl of 10 mM Tris-HCl (pH 7.5), 150 mM NaCl, 2 mM MgCl_2_, 5 mM DTT, and 50 units of Benzonase. The sample was then incubated at 37°C for 30 min. After 30 min, 225 μl of 0.1M Na_2_CO_3_ and 2 M Urea was added and incubated at 4°C for 10 min. Next the samples were centrifuged at 50,000 rpm at 4°C for 10 min. The supernatant fraction was collected, and the pellet was rinsed in HN buffer, and re-centrifuged. The pellet and supernatant fractions were incubated at 95°C for 10 min with 5X SDS running buffer plus BME, then subjected to SDS-PAGE and immunoblotting with the indicated antibodies. For quantification in Fig 5B, bars represent the relative values of L2-FLAG in the pellet fraction normalized against the L2-FLAG level in the pellet fraction of scrambled siRNA treated cells. The values were further normalized against the BAP31 band intensities. Bars represent the means ± SD of at least three biological replicates. Statistical significance was determined using the student’s *t* test.

## Supporting information

S1 Fig Additional data characterizing a role of p120 during HPV16 infection (related to Fig 1). **A.** Purified WT HPV16.L2F (GFP-S), WT HPV16.L2F (Luc), WT HPV5.L2F (Luc), and WT HPV18.L2F (Luc) used in this study. Samples were subjected to SDS-PAGE and staining with Coomassie blue. The positions of L1 and FLAG-tagged L2 are indicated. **B.** HeLa cells infected with WT HPV16.L2F (Luc) for 6 hrs were lysed and the resulting extract subjected to immunoprecipitation using a FLAG antibody, or an equal concentration of IgG control antibody. The precipitated material was analyzed by SDS-PAGE and immunoblotting using the indicated antibodies. Samples labelled input were not immunoprecipitated. **C.** HeLa cells were infected with HPV16.L2F (Luc). 2.5 hpi, cells were lysed and the resulting extract was subjected to immunoprecipitation using an antibody against p120, or an equal concentration of IgG control antibody. The precipitated material was analyzed by SDS-PAGE and immunoblotting using the indicated antibodies. Samples labelled input were not immunoprecipitated. **D.** HeLa cells transfected with the indicated siRNA were treated with trypan blue 72 hours after transfection to stain dead cells. Data represent the mean ± SD of three independent experiments. **E.** HeLa cells were seeded at equal amounts and transfected with the indicated siRNA. 72 hours after transfection, cells were harvested and the total number of cells per condition were counted by hemocytometer. Data represent the mean ± SD of three independent experiments. **F.** HeLa cells transfected with the indicated siRNA were infected with SV40 and subjected to immunofluorescence staining using an antibody against SV40 large T antigen. Data are the percent of cells expressing large T antigen, as assessed by fluorescent microscopy, normalized against SV40-infected cells treated with scrambled siRNA and represent the mean ± SD of three independent experiments. **G.** C33A cells transfected with the indicated siRNA were infected with or without WT HPV16.L2F (Luc). 48 hpi, luciferase activity was measured from the cell culture media. Graph shows luciferase activity normalized against scrambled siRNA-treated cells with and without HPV. Data represent the mean ± SD of at least three independent experiments. The immunoblot reveals the extent of p120 depletion. **H.** As in G, except HaCaT cells were used.
(TIF)

S1 Table Potential HPV16-interacting host factors. Full results from Inoue et al., 2018 of L2 immunoprecipitation-mass spectrometry performed on samples of HeLa cells infected with WT HPV26.L2F for 16 hrs or uninfected cell extract incubated with purified HPV16 PsV (called mock-infected in table). The total number of peptides corresponding to the mock or HPV-infected cells are bolded. The results for p120 are highlighted in green.
(XLSX)
