# Supplementary figures and images for: p120 catenin recruits HPV to γ-secretase to promote virus infection
Source: PLoS Pathog. 2020 Oct 21;16(10):e1008946. doi: 10.1371/journal.ppat.1008946 (PMC7577436; doi:10.1371/journal.ppat.1008946)

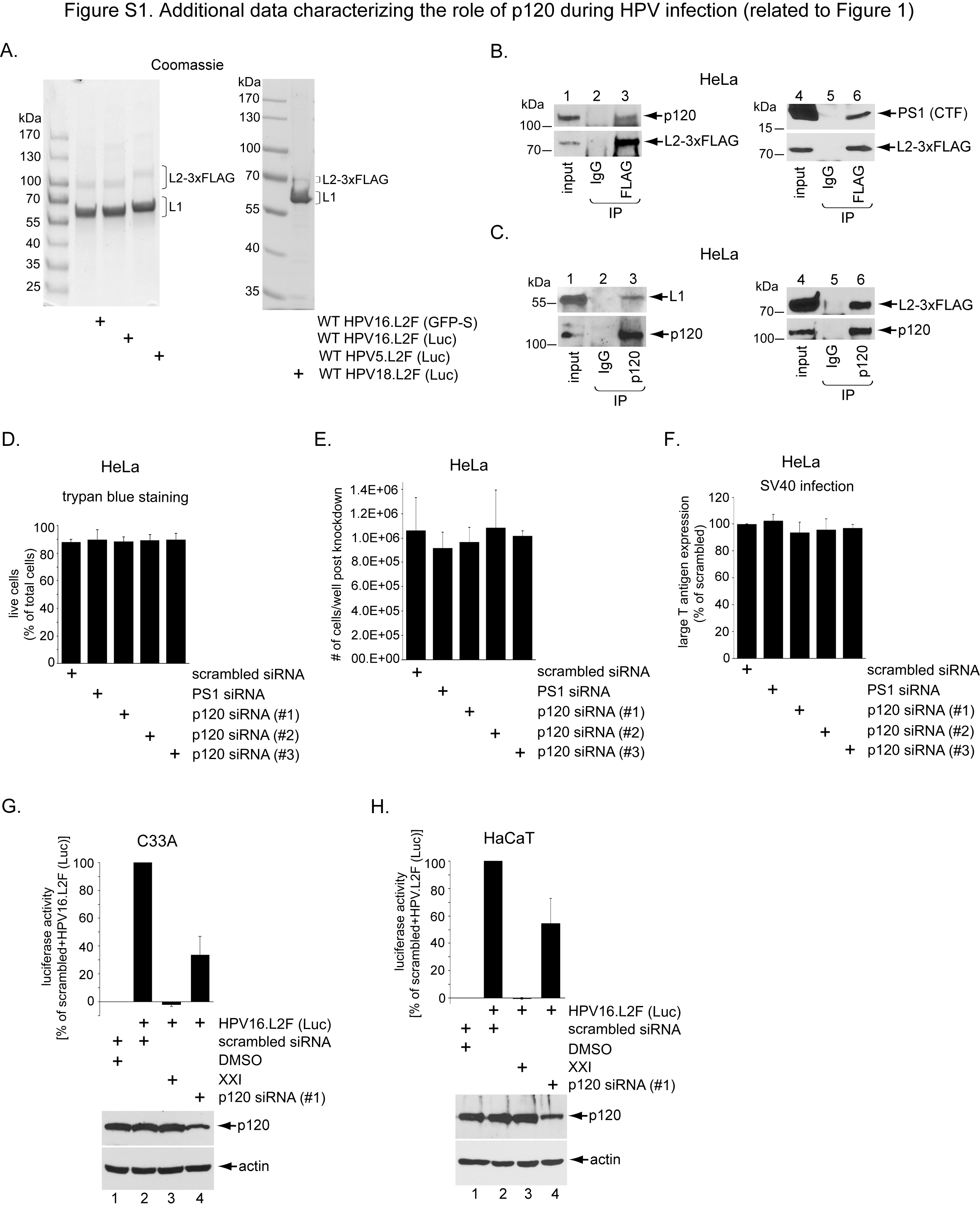

Supplement: S1 Fig — A. Purified WT HPV16.L2F (GFP-S), WT HPV16.L2F (Luc), WT HPV5.L2F (Luc), and WT HPV18.L2F (Luc) used in this study. Samples were subjected to SDS-PAGE and staining with Coomassie blue. The positions of L1 and FLAG-tagged L2 are indicated. B. HeLa cells infected with WT HPV16.L2F (Luc) for 6 hrs were lysed and the resulting extract subjected to immunoprecipitation using a FLAG antibody, or an equal concentration of IgG control antibody. The precipitated material was analyzed by SDS-PAGE and immunoblotting using the indicated antibodies. Samples labelled input were not immunoprecipitated. C. HeLa cells were infected with HPV16.L2F (Luc). 2.5 hpi, cells were lysed and the resulting extract was subjected to immunoprecipitation using an antibody against p120, or an equal concentration of IgG control antibody. The precipitated material was analyzed by SDS-PAGE and immunoblotting using the indicated antibodies. Samples labelled input were not immunoprecipitated. D. HeLa cells transfected with the indicated siRNA were treated with trypan blue 72 hours after transfection to stain dead cells. Data represent the mean ± SD of three independent experiments. E. HeLa cells were seeded at equal amounts and transfected with the indicated siRNA. 72 hours after transfection, cells were harvested and the total number of cells per condition were counted by hemocytometer. Data represent the mean ± SD of three independent experiments. F. HeLa cells transfected with the indicated siRNA were infected with SV40 and subjected to immunofluorescence staining using an antibody against SV40 large T antigen. Data are the percent of cells expressing large T antigen, as assessed by fluorescent microscopy, normalized against SV40-infected cells treated with scrambled siRNA and represent the mean ± SD of three independent experiments. G. C33A cells transfected with the indicated siRNA were infected with or without WT HPV16.L2F (Luc). 48 hpi, luciferase activity was measured from the cell culture media [file ppat.1008946.s001.tif]
